# Turning to Peers: Integrating Understanding of the Self, the Condition, and Others’ Experiences in Making Sense of Complex Chronic Conditions

**DOI:** 10.1007/s10606-016-9260-y

**Published:** 2016-08-17

**Authors:** Aisling Ann O’Kane, Sun Young Park, Helena Mentis, Ann Blandford, Yunan Chen

**Affiliations:** 1 University College London, London, UK; 2 University of Michigan, Michigan, MI USA; 3 University of Maryland, Baltimore County, Baltimore, MD USA; 4 University of California, Irvine, Irvine, CA USA

**Keywords:** Chronic condition, Exploratory search, Healthcare, Information seeking, Open-source, Self-management, Self-care, Patient knowledge, Peer interactions

## Abstract

People are increasingly involved in the self-management of their own health, including chronic conditions. With technology advances, the choice of self-management practices, tools, and technologies has never been greater. The studies reported here investigated the information seeking practices of two different chronic health populations in their quest to manage their health conditions. Migraine and diabetes patients and clinicians in the UK and the US were interviewed about their information needs and practices, and representative online communities were explored to inform a qualitative study. We found that people with either chronic condition require personally relevant information and use a broad and varied set of practices and tools to make sense of their specific symptoms, triggers, and treatments. Participants sought out different types of information from varied sources about themselves, their medical condition, and their peers’ experiences of the same chronic condition. People with diabetes and migraine expended great effort to validate their personal experiences of their condition and determine whether these experiences were ‘normal’. Based on these findings, we discuss the need for future personal health technologies that support people in engaging in meaningful and personalised data collection, information seeking, and information sharing with peers in flexible ways that enable them to better understand their own condition.

## Introduction

There has been a push in recent years towards moving care outside clinical settings and giving patients more responsibility in order to contain costs and promote independent living (Mort et al. 2013), increasingly through the use of personal health tools. With new wireless, networked, and sensor-based advances, the choice of self-management practices, tools, and technologies has never been greater (Randell et al. 2010). Personal health tools have been the focus of much research in HCI (Nunes et al. 2015) and CSCW (Fitzpatrick and Ellingsen 2013), with some positive results in terms of behaviour change and emotional support. Personal health informatics systems for tracking one’s personal health variables have been attributed to promoting self-reflection and awareness in self-care (Aarhus et al. 2009). Online health communities and forums have been shown to empower patients to learn from each other (Hartzler and Pratt 2011) and provide social-emotional support to patients who may not be able to obtain such support in their offline relationships (Preece 1998). Similarly, Internet-based social networks have been promoted as an effective way for patients to gain emotional support and help from family and friends (Skeels et al. 2010; Pols 2012).

However, compared to the care of acute conditions, “chronic disease care is fundamentally different” (Funnell and Anderson 2000, p. 1709) in terms of requiring on-going information seeking for managing conditions over time, which has implications for the design of personal health tools. Some chronic conditions have triggers, symptoms and treatments that can differ significantly for each individual and the idiosyncrasies of each condition makes self-management a daunting task. Despite major differences between chronic conditions, there are commonalities between their characteristics and their challenges (Wagner et al. 2001). Two such conditions are diabetes^1^ and migraine^2^; although these chronic conditions differ significantly, both are idiosyncratic and demand varied self-management practices and there are similarities in many of the personal health tools available for each.

As well as being idiosyncratic, these conditions are also complex: they require a concentrated effort to identify symptoms and triggers that are *unique* to a person’s illness experience. Discovery is often through trial and error to identify personally appropriate data collection and treatment plans. Consolvo, McDonald, and Landay suggest designers should be aware of how lifestyle change “pervades everyday life” (Consolvo et al. 2009, p. 414)*.* Maitland and Chalmers (2011) suggest that design focus should be on how health tracking technologies fit into people’s everyday lives. Ballegaard, Hansen, and Kyng also suggest that health technology design should concern “not just the matter of fixing a health condition, more importantly is the matter of sustaining everyday life as a whole” (Ballegaard et al. 2008, p. 1807). Kanstrup working with families with a member who has diabetes, suggests that “designing for the real world calls for engaging with peoples’ real problems” (Kanstrup 2014, p. 49)*.* These are important concerns for conditions that require active participation by people outside the clinical setting, such as diabetes where 95 % of care is self-care, making people “more than passive recipients of medical expertise”(Funnell and Anderson 2000, p. 1709).

Self-validation of illness experiences is important for people with these chronic conditions as they have different symptom representations that can lead to uncertainty in self-management practices. These individual health differences lead to the need to acquire personal expertise in chronic illness care that is specific (and practicable) to their illness experience (Prior 2003; Pols 2013). Moreover, there are specific “physiological, personal and social activities that impact the process of individual disease management” (Chen 2010, p. 253). This motivates our research on how people engage in self-management and information seeking from varied sources. The aim of the research reported here is to inform the development of personal health tools that support people’s self-validation of illness experiences when they are faced with self-managing idiosyncratic and complex chronic conditions.

Building on previous research on information seeking practices in healthcare, this paper outlines data and health management information seeking practices related to diabetes and migraines. Taking a bottom up approach to understanding current practices, we report on personal information seeking practices of, and tools used by, people managing these conditions. Based on early discussions in which findings on the management of these chronic conditions were compared and similar issues were found, we cross-compared the two data sets to identify overarching themes for chronic disease management that had emerged independently and had similarities despite the differences between diabetes and migraine.

We found that people engage in exploratory searches to better understand what is ‘normal’ in terms of the manifestation of their particular symptoms, the variables they should be tracking, and the effects on their lifestyle and medication adherence. People’s needs to live “life as normal” (Robinson 1993, p. 6) have been discussed in research with regards to medical conditions outside the CSCW domain (Bartlett 2011; Genuis 2012), including around chronic conditions (Robinson 1993). Bartlett laments that conventional wisdom leads people to believe “normal is good, abnormal is bad” (Bartlett 2011, p. xi). By ‘normal’ we do not mean ‘typical’, but rather ‘acceptable’, which suggests that what a person has discovered about their own health experience is close to what is experienced by others (Genuis 2012; Penrod et al. 2012). This is described by Penrod et al. (2012, p. 181): ““Seeking normal” refers to processing of available information that aims to recreate a sense of pattern in the midst of chaos”. This, in part, is to validate their unique illness experience and ultimately feel they have gained some control of their condition.

These findings highlight a gap in the current technology solutions for personal health management of chronic conditions: namely, to support this individual search for ‘normal’ by contextualizing one’s own observations and experiences by relating them to experiences of others. An opportunity to close this technology gap through further research is presented: a turn towards personal health systems that support information seeking from varied repositories and peers to reduce current effortful practices and help people situate their experience against a backdrop of ‘normal.’

## Background

With the growing use of smartphones that can connect to the Internet, and mobile medical devices that are connected to these smartphones (Tran et al. 2012), new interactive systems have been designed to assist in self-management practices for chronic conditions such as diabetes and migraine. To set the context for the work reported here, we review related work on information seeking for health, chronic condition self-management, and technologies connected to the self-management of chronic conditions.

### Information seeking for health

Information seeking is a process that is well studied in the HCI and CSCW domains and its importance is recognised in the healthcare space as well: it is defined as the attempt to gain information in both human and technological contexts (White and Roth 2009). Health information sharing for chronic care has been discussed in the CSCW community; for example, Bansler and Kensing (2010) highlight the importance of delivering information structures that cross boundaries, both institutional and professional. Health information seeking and health information needs have also been studied extensively in the information sciences community (Case 2006; Fisher and Julien 2009). In addition, new information seeking practices, focusing on seeking out the experiences of fellow patients, are being further investigated for chronic conditions (Wicks et al. 2010). Pols, looking at “Care at a Distance,” describes the use of tools such as webcams to connect people to share “a set of physical, emotional and practical variables that they share” (Pols 2012, p. 70). This has been identified as an important behaviour as people can seek out others with similar experiences who “are very happy to support each other, all having been in the same position themselves” (Pols 2012, p. 69). The move to greater personal responsibility for people’s own health management has led to changes in information seeking practices among people with chronic conditions in addition to more traditional means such as books, magazines, TV programs, etc. In 2012, more than half of U.S. adults reported looking online for health information, with 35 % self-diagnosing a condition and 16 % trying to find others with the same health concern (Kuehn 2013).

White and Roth discuss the rise of more open-ended information seeking practices involving exploratory search, which they define as “open-ended, persistent, and multifaceted, and information-seeking processes that are opportunistic, iterative, and multitactical” (White and Roth 2009, p. vi). They discuss the move away from the traditional model of query/response information seeking towards a model that supports human resourcefulness. This non-linear information seeking model has been supported in HCI research, where Adams and Blandford identify the problem that “no sooner have users’ needs been identified and supported than they change” (Adams and Blandford 2005, p. 160) They discuss the temporal nature of the information journey these users take, which has parallels to the trajectory of healthcare (Strauss 1975) and the temporal nature of information seeking in clinical settings (Reddy et al. 2006).

Information seeking supports people gaining more extensive knowledge about symptoms leading to better self-care practices, independent of how controllable the chronic illness is (Felton and Revenson 1984). Mishel (1990) suggests that uncertainty in illness can be reframed as an opportunity instead of a danger, so that patients engage with their personal health. This links to the open ended nature of exploratory search, which has been observed in people’s health information queries (Cartright et al. 2011) and found to broaden the scope of information explored, leading to better learning (Chin and Fu 2012). These investigations into people’s exploratory search for health information inspired the investigation of the exploratory information seeking experience of people who are managing a complex chronic condition, either diabetes or migraine.

### Chronic illness self-management

Like many other chronic conditions, diabetes and migraine place a high responsibility on a person to monitor and manage their disease as not doing so could have serious consequences. For instance, for both Type 1 (T1D) and Type 2 (T2D) diabetes where the pancreas does not produce sufficient quantities of the hormone insulin needed for the digestion of glucose (Davis et al. 2015), low blood sugar levels (hypoglycemia, or ‘hypos’) can lead to immediate health concerns including feeling physically ill and proceeding to unconsciousness or a diabetic coma. On the other hand, excess levels of blood sugar (hyperglycemia or ‘hypers’) can eventually lead to complications such as eye, foot, kidney, and heart disease. Migraine, a type of recurrent throbbing headache, typically does not lead to death, but can be accompanied by nausea, vomiting, sensitivity to light, and/or disturbed vision (Dahlof and Solomon 1998). Many patients are unable to move during migraine attacks and have to manage their triggers and wait until the symptoms disappear to resume their normal activities. Consequently, management activities are of the utmost importance to those dealing with these chronic conditions in order to maintain wellness and reduce the impact of their condition on their everyday life.

For diabetes, symptoms of hypos or hypers can vary significantly and feel very different between people, triggers of high and low glucose levels can vary by person and by a range of external conditions, and treatments such as timing of dosage, the type of insulin used or the amounts needed can vary by person and change over time (Mol and Law 2004). The information on these condition specific variables can be overwhelming to record and deal with over time (O’Kane et al. 2013). People with T1D must estimate medication doses to give via injections or a programmable insulin pump by tracking factors ranging from personal lifestyle (e.g. diet, exercise, sickness and stress) and their current blood sugar level reading, to the weather’s influence on blood sugar levels and plain instinct. In comparison, many people with T2D, have a more standardized routine of ingested medication, but are still required to conduct ‘pattern recognition’ (Linekin 2002) to account for environmental factors, plan their exercise, and ‘count carbs’, sometimes also using a blood glucose monitor to help them monitor their levels (Chen 2010). Some people with T2D have to engage in similar practices to people with T1D, such as giving injections.

There are many major differences between diabetes and migraine in terms of daily practices, consequences, and medical technologies available; however, there are striking similarities in how idiosyncratic these conditions are. Like diabetes, the migraine condition does not have a homogeneous set of symptoms, triggers, or treatments where a one-size-fits-all self-care practice is sufficient. Symptoms vary widely with some people having hours of occasional and mild attacks such as dizziness and pulsing pain, while others suffer frequent attacks with severe pain, vomiting and physical disability for up to a week. It is often difficult to distinguish whether a symptom has resulted from migraine or another condition, or is a side effect of a medication (Bigal and Lipton 2006). As for diabetes, this requires the person with migraines to monitor their management practices in order to reduce the impact the illness has on their life. These challenges in the diagnosis and treatment of migraines can lead to frustration, a lack of satisfaction, and discontinued clinical consultation (Lipton et al. 1998).

Careful management practices can help prevent the condition from significantly impacting a person’s everyday life. However, the sheer amount of data it is *possible* to collect and analyse from a range of different sources can become overwhelming and often does not seamlessly fit into one’s experience of the illness. This self-tracking of data can also be considered a communicative process, with Lomborg and Frandsen (2015) suggesting it has three dimensions: communication with the system, the self, and social networks of peers. An increasing number of systems are being made available that aid the self-tracking of people with chronic conditions in an attempt to deal with the overwhelming amount of data associated with self-care.

### Technologies for self-management

There have been prior studies focused on monitoring and collecting personal health information to support crucial aspects of chronic care management, such as tracking disease progress and sharing collected information with clinical providers (Mamykina et al. 2008; Yun et al. 2012). For both conditions, there are general healthcare tools available, such as Microsoft Healthvault,^3^ a web and mobile based system that allows users to track their condition, with some connections to medical technologies, and also the ability to share information with medical professionals – essentially, personal health records. Likewise, patientslikeme.com is a web based system that allows patients to learn about their condition, track medical information and connect with patients with similar conditions using their specific platform (Wicks et al. 2010). Researchers in HCI, CSCW and medical informatics have shown that these tools can support maintenance activities by aiding self-reflection (Franklin et al. 2008; Årsand et al. 2010; Cafazzo et al. 2012; Chatterjee et al. 2012).

For diabetes, there are a large number of commercially available systems (Mendosa 2016) including websites and apps that allow information seeking on different foods, data entry, and synchronization of data (Aarhus et al. 2009). For migraine sufferers apps are less common; however, mobile electronic diary monitoring has been found to be more useful and positive than traditional paper-and-pencil methods in terms of user-friendliness, absence of burden, and perceived support for migraine attack prevention (Sorbi et al. 2007), and online training programs before clinical visits have been found to have positive impacts on clinician-patient communication (Sciamanna et al. 2006).

Although online and mobile health tools are designed for tracking related events, improving the quality of the clinical consultation, and informing the public about their health conditions, *they require or assume that people with chronic conditions already know their symptoms, triggers, and treatments*. Their design assumes that the data presented is self-evident – that people will be able to interpret the stand-alone health data they collect despite its complexity (Mamykina et al. 2006). Moreover, the technologies and their associated research focuses on health outcomes, and not on quality of life. Naci and Ioannidis lament that “Most medical research focuses on disease rather than health. Yet people are interested predominantly in health and wellness” (Naci and Ioannidis 2015, p. 121). Beyond assisting in self-management, there is also potential for technologies to aid the emotional aspects of chronic care self-management, and there are cost-savings connected to supporting psychosocial needs in chronic care (Sobel 2000). This motivates our research on self-care and how people engage in information seeking from different sources in order to better understand and manage their illness experience. It is essential to first obtain a thorough understanding of people’s health information seeking behaviours and needs, as exhibited in real condition self-management processes.

We performed two exploratory studies of people’s information seeking related to the self-management of diabetes and migraine using a semi-structured qualitative approach (a flexible and pragmatic approach to qualitative research that has been used in health related research) (Blandford 2013). We present a qualitative analysis of the information seeking practices uncovered that points to people with idiosyncratic self-management practices persistently looking to validate their experiences by comparing their own symptoms, triggers, and outcomes with what the medical community suggests and what their disease peer group experiences, based on both their personal condition and their personal preferences. These practices are effortful, and exploit a range of sources. Based on our findings, we identify a requirement for future personal health tools that support people in self-validation and determining whether what they are experiencing is ‘normal’ by contextualising their own experiences through relating their own recorded data with established medical knowledge and also the experiences of peers.

## Method

Two exploratory qualitative interview based studies were carried out – a diabetes study in the United Kingdom and migraine study in the United States, with both also exploring a selection of worldwide online communities for each condition. We compared these two chronic illness populations because both require a rich understanding and interpretation of personal data for self-management of a complex chronic condition. The data collection activities involved in both studies are summarized in Table 1. The focus of analysis was on the self-management practices of people who are dealing with unique triggers, symptoms and treatments.

**Table 1:** Data collection of the migraine and diabetes studies.

	Patients and online communities	Clinicians
Diabetes study	9 interviews, one group interview (20 participants), and 3 interviews with patient bloggers	6 interviews
Migraine study	12 interviews and a review of 2 online forums	2 interviews

### Diabetes data collection

Data on diabetes data management practices was collected with support from UK hospitals’ diabetes service groups. An initial review of online diabetes resources such as blogs and technologies provided an understanding of the technology domain, and attending local support groups gave an understanding of the wide variations in diabetes management practices. Based on insights from the initial exploration, interviews were conducted in an open exploratory manner focusing on current management practices with diabetes specialist clinicians and people self-managing T1D or T2D. The focus of the questions was on people’s personal experiences of gaining, managing, and sharing health information, with technology or otherwise.

Interviews were initially conducted with three diabetes bloggers who were recruited based on their experience in the Diabetes Online Community (DOC) (Hilliard et al. 2015) and two people known personally to the researchers. We then conducted a further seven telephone interviews with people with T1D or T2D recruited from online advertisements, attending urban and suburban diabetes support groups, and physical advertisements in grocery stores. In addition to these 12 interviews, we were invited to conduct a group interview with 20 people at a diabetes support group that we had previously attended. The group interview was organised by asking questions of the members and allowing them to discuss the answers among themselves. This ensured a wide range of interviewees with varied experiences and backgrounds, including those with T1D since childhood, two with T1D onset in their early thirties and forties, elderly people with T2D diabetes, and one with T2D diabetes onset in their late twenties.

We interviewed six UK based diabetes health specialists: two senior diabetes specialist clinician consultants, a midwife specializing in diabetes care during pregnancy, a diabetes specialist nurse, a diabetes specialist podiatrist, and a diabetes specialist dietician. They were recruited from diabetes services groups in two hospitals in Eastern England through the head of one of the services groups.

Informed consent was obtained and interviews were held in person and over the phone in a semi-structured format. Each interview concluded when the interviewer felt a clear picture of the participant’s perspectives on medical information seeking and sharing had been established; all interviews lasted at least half an hour. The interviews, including the group interview, were all recorded and transcribed for data analysis.

### Migraine data collection

To obtain a basic understanding about the migraine condition and associated self-management practices, we began with a pilot study on two online forums to understand the common concerns of people with migraines.

Mymigraineconnection.com was chosen because it had the largest number of posts (over 20,300 posts directly related to migraine symptoms, triggers, medications, support, etc., as of November 2011) among migraine forums and because the conversations there focused on practical information sharing rather than emotional support like some other communities. Additionally, WebMD.com was chosen since several interviewees referred to it at the beginning of our study. We chose to study only the community forum section because our research intention was to study interactions among peers. We randomly selected threads from the topics ‘medication & treatment’, ‘doctors & clinics’ and ‘discussion’ for our analysis.

During the first two months, we initially analyzed information from two online communities by using open coding (Corbin and Strauss 1990) in our pragmatic semi-structured qualitative approach (Blandford 2013). Three researchers looked for salient concepts and their properties (i.e., characteristics), and identified themes (i.e., communication with clinician, coping strategies). Through the pilot study, it was noticed that very few people with migraines actually chose to use the online portals. To further explore these findings, we conducted interviews with people with migraines and primary care clinicians to gather a broader set of data about the behaviours exhibited in people’s real lives, specifically focusing on issues such as people’s personal experiences with migraine attacks, including their symptoms, triggers, treatments, and coping strategies; how people communicate about their migraine with others in their social circle; and how people seek and find migraine-related information.

We performed contextual interviews with 12 urban and suburban participants in their own homes – the place where most migraine management activities occur. To recruit participants, we sent the study announcement broadly through various mailing lists, posted fliers, and also used a snowball sampling method. As in the diabetes study, we recruited a diverse set of participants with migraine conditions that varied greatly, from very severe cases with many complications to non-severe conditions; from managing the disease at home to dealing with it at work or on other occasions.

We also interviewed two primary care physicians to understand clinicians’ attitudes about migraine management over the telephone. We involved primary care physicians because patients often visit them as their first step in treating migraine.

Informed consent was obtained and patient interviews lasted approximately one hour. The clinician interviews were conducted over the phone and lasted for half an hour. All interviews were recorded and transcribed for data analysis.

### Data analysis

Thematic analysis (Braun and Clarke 2006) was chosen to systematically analyse the combined data whilst also allowing us to tap into latent themes that manifested in the data collected. A three-step approach was used for coding the data. Coding was organized first around the broad theme of ‘information seeking’ in the larger data set (the diabetes study) followed by iterations of analyses through both data sets focusing on commonalities and contradictions. The evidence that people were seeking out what is ‘normal’ in these complex conditions led to a further focus on themes related to the complexity of information needs for each condition, current information seeking practices, and strategies for overcoming current information gaps. Finally, this coding allowed deeper analysis of the commonalties and differences in personal information seeking, seeking information about the condition itself, and seeking out information from peers. As well as ensuring that we included people with variation in experiences, we aimed to pragmatically maximize the validity and reliability of findings by triangulating data (Golafshani 2003) collected by different means (as described above) and relating findings to prior literature. The emergent themes motivated a review of the design of personal informatics systems for chronic care management.

## Findings

Although people with migraine and those with diabetes have to deal with substantially different experiences in their care management, we found that both conditions led to participants seeking information about themselves, about the conditions in general, and about other similar experiences from their peers from a wide variety of sources in a persistent effort to attain a personally relevant account of ‘normal’.

### The struggle to understand if personal data is normal

Diabetes and migraine are both idiosyncratic, so people with these conditions experience a range of triggers and symptoms, and a wide variety of treatment plans. Each illness management case is unique and personal – in other words, there is no average patient. Each person needs to be aware of and manage their unique triggers and symptoms, and conduct some experimentation with treatments for the care of their condition. This means that they must have awareness of and reflect on their conditions and their experiences. Our participants suggested the number of variables to attend to can make reflection an overwhelming task.

“I’m consciously or subconsciously thinking of my blood sugars, the last time I took insulin, where my blood sugars are going, who I am caring for at that moment, what kind of ranges am I willing to fall into, during the course of these activities. You’re talking about the backburner, it’s kind of on all burners regardless of what’s cooking, you know?” – Diabetes Blogger 2

Migraine management, like diabetes, is not straightforward, with combinations of symptoms that can be unique for an individual and treatments, including medications, which work differently for different people. Since migraine symptoms are often related to other health issues such as brain tumours, strokes, or side effects of other medications, tracking personal triggers and medication can become difficult. Similar to Mol and Law’s (2004) discussion of dealing with T1D hypos involving “*measuring and feeling*” in order to diagnose and treat, and repeat if correction is needed, some migraine management practices include trial and error to test and track outcomes, which can be a complicated and surprising process.

“What works surprisingly well for me (and please read this as FOR ME and ME ONLY) is a combo of [anti-inflammatory drugs] and a megadose of Benadryl. If I can take this during the aura, for some reason it dulls down my [central nervous system] enough to keep the migraine to a minimum. We aren’t sure exactly why this works for me, since they aren’t really abortive meds. It took a lot of trial and error, but it got figured out.” - Migraine Online Forum 1

The personal nature of both these conditions led to varied needs for the collection of personal health information and varied practices, including writing notes but also just remembering relevant information. The variables associated with both conditions can be numerous, which can make ‘objectifying health’ (Pols 2013), tracking and reflecting on these variables, a complicated, on-going task. Participants mentioned that it could cause lack of confidence in their techniques, leading them to question themselves and their experience of the condition in relation to their everyday lives.

“You’re always thinking what have I had to eat? How much insulin have I had? When will I next eat? What will my insulin requirements be then? How do I feel now? Do I feel like I’m having a reaction? Am I going to have a reaction? What exercise…? […] It’s rarely out of your mind. Because if you get a bit hot, you should- oh god. Is that the beginning of a reaction?” - Diabetes Patient 12

“I write down when it starts with like the mild headache… Really, the reason I wanted to start writing them down was to see how often are they and how bad are they. My doctor didn’t really ask me to do but this is partly because I’m like what’s going on, how can I make this stop?” – Migraine Patient 12

The numerous variables and ambiguous relationship to outcomes can lead to exhaustion. Diabetes is often referred to as a relentless condition and this leads to issues in varied data capture practices, as ‘burn-out’ can occur (Polonsky 1999). While numerous techniques for tracking the variables associated with the condition are available, participants mentioned that they could lose motivation over time to use them, even with technology aids.

“That’s been very difficult to capture all that information, so I’ll go in spurts where I try to using some kind of a record keeping tool. They range anywhere from a simple Excel spreadsheet to software package that someone else has put together, but I have never been really satisfied with any of them. I don’t feel like any of them allows me to easily capture all of the information. But part of that is not necessarily the fault of the software or the tool that I’m using. I think there’s a fair amount of burn out that happens trying to capture all of that information. It’s an incredible load.” – Diabetes Blogger 1

For migraine sufferers, since attacks can be very unpredictable and they suffer from pain during attacks, they experience difficulties in tracking their condition at the very moment they would benefit most from recording this information. Participants and online forum members often reported difficulties in tracking and timing.

“Well, the doctors told me that I should have a calendar and just write down when I have a headache. But, I’m just not finding the right time for writing down because it mainly starts when I’m in school, so I don’t really have a tool to record. […] and I sometimes don’t even keep track of the time.” – Migraine Patient 4

The idiosyncrasies of the symptoms, triggers and treatments of both conditions led our participants to want to know what are the ‘right’ variables, the acceptable number of variables, or even the most significant or useful variables for their management practices. Some participants described wondering how their personal variables compared to others’ variables, for example whether other people shared the same migraine food triggers. This resulted in participants and online forum members seeking out information from their peers and expressing worry about how their illness experiences compared to others’.

“I have a lot of the usual triggers […] but the one I struggle to keep in check the most is onions... I’ve never seen it listed as a trigger anywhere so I was wondering if anyone else has this problem? It’s just the hardest food to avoid because it is in EVERY convenience food going.” -Migraine Online Forum 2

For diabetes and migraine participants, the personal nature of the conditions requires tracking of often unique health variables, in various ways that are suited to that individual. The variety in triggers that cause migraines or that lead to high and low blood sugar means that both groups of participants were tracking and reflecting on a myriad of variables such as physical activities, diet, stress, mood, weather, etc., which was described as sometimes overwhelming. The differences in the symptoms in either condition means that participants had to reflect on how they feel and try to relate this to the unique triggers they have recognized. The differences in the treatment of these conditions can also vary widely and finding the right balance between what is prescribed to them and what they have recognized as personally effective can be difficult. These practices lead to a patient needing to record information in the moment, through personal and varied means such as remembering, writing a note, or sometimes using a technology aid; this supports earlier findings in personal informatics on the personal toll of tracking and reflection on personal data without appropriate analytical tools (Li et al. 2011). However, in order to ensure the usefulness of the variables recorded or remembered, patients look to reduce or narrow the set of variables that they attend to. Discovering this set, however, takes much effortful information seeking from various sources and comparison to their own experiences.

### Medical information sources are not adequate for validating normality

Although managing all of this personal information can help a person identify what is a typical illness trajectory for them, it does not help them to contextualize and interpret their experience as ‘normal’. Instead, they seek out other sources of information to compare against their personal results. Although this could be considered an established information journey for a person (Attfield et al. 2006), it is generally not a straightforward path, entailing significant data capture and analysis for health management for those with complex chronic conditions such as diabetes and migraine. There are many sources of information available, and the amount of information about these complex conditions is vast, and therefore time consuming to assimilate in order to aid self-management.

Accessing medical information from the traditional source of this knowledge, clinicians, can be problematic, for reasons such as time concerns. Getting information from clinicians can be a time consuming and effortful process, and usually only occurs during appointments and in a small number of other communication opportunities. Even at the appointment, some clinicians will not always spend the time to educate or share health information with patients, to the frustration of some participants.

“If I ask when they are sitting there in front of me, they’ll share my cholesterol, blood work, and all that, and my heart rate. But if I don’t ask, they often don’t share it […] they just, just think their patients are too dumb to understand what the numbers mean or that we don’t care. A little frustrating.” – Diabetes Blogger 3

The lack of information from clinicians is a recurring problem, so patients are looking elsewhere for medical information. Sometimes sources that may not be the most reliable or written in the interests of the patient can be easier to access, and are therefore relied upon. This in turn can cause frustration in the relationship between clinicians and patients, as voiced by some of our clinicians who have to clarify incorrect or inappropriate information and help patients situate their personal disease management within sometimes fleeting health fads that gain attention from the media. The vast number of sources to review can take up precious time in appointments.

“They are constantly saying about the Daily Mail or things that they read. […] Loads of things they read off BBC News, website and things. They’ll always bring that. The latest fad is always brought to appointments.” – Diabetes Dietician

Furthermore, because of the amount of data associated with the conditions, our participants suggested that it is possible that patients can get overwhelmed with the amount of medical information available to them and they may not understand the medical language. Becker (2004) concluded that many online health resources would require a college education to understand the medical information. This was similarly noted by one clinician who discussed an online health management system aimed to educate and inform patients, which ultimately failed because of the overwhelming amount of information on the site.

“I mean that’s the thing with the Health Spaces, we did feel like it was very useful and people weren’t even using it. They found it too complicated to use.” – Diabetes Dietician

Unfortunately, some patient participants suggested that they cannot trust their clinicians to provide valid and up to date information, which is in line with related research (Attfield et al. 2006). In these cases, they look to other sources of medical information, often turning to medical resources on the Internet, such as WebMD.com. This can be seen particularly when someone is first diagnosed and they immediately look to Internet sources to supplement what they think is a lack of information from their clinicians.

“When they did actually release me I was basically kicked out. I was given a bag full of supplies and said… basically told me to ‘inject this much at this point in the day and inject this much at this point in the day, bye!’ I was like ‘oh, ok.’ And the same sort of thing, within the first couple of days after I got home […] I read up all about it. I thought well, ‘how does it work?’ I mean it can’t make sense that you just inject the same every time […] So I thought, let’s look at the science behind this.” – Diabetes Patient 2

We found that, in addition to participants not trusting their clinician’s level of expertise about these complex conditions, they were also not satisfied with comparing medical information from websites with their personal health information; some participants were looking towards patient generated sources of information to get a better grasp on what was normal. When a prescribed migraine medication does not resolve their symptoms effectively or immediately, patients can turn to others to look for alternatives.

“Maybe I am just being impatient but I just don’t think the meds are working as well as they could be. With so many options out there what should I try next? […]. I am sure my doctor will have some sort of idea but I guess I want to be educated and have a plan of action before I go in.” – Migraine Online Forum 1

In the quest to contextualize and situate their own condition’s manifestations, many of our participants sought out further medical information. The traditional source of this information is from their medical practitioners, but this information seeking process can be met with time constraints and trust issues. When looking through repositories of health information, participants reported that patients can feel overwhelmed by the medical terms used and can turn to sources that might not be written in their best interests. Even when people were able to get a handle on the medical information about their chronic condition from their preferred source(s), they still needed to compare their personal information to the general information about the condition, and even go further to compare their chronic condition experiences to their peers’ in order to understand what is ‘normal’ and where they fit in. Participants had their own preferences for seeking medical information and used varied methods to achieve this (e.g. contact with clinicians, WebMD, etc.), including turning to their peers.

### New opportunities in validating normality through turning to peers

Building on understanding general medical information about their condition to aid their self-management practices, our participants are also trying to place themselves amongst the larger illness community to understand their health data and illness experiences. When looking at how patients share information and support each other, Civan and Pratt (2007) believe peers provide informational, emotional, and instrumental support for understanding, interpersonal connectedness, and practical assistance, which has been described as “*together-management*” (Pols 2012, p. 75). We found that participants were looking to their condition peer group, i.e. other people who share similar illness experiences.

There were common themes for both of these chronic conditions with regards to validating illness experiences with peers rather than clinicians. Similarly, Pols describes a furniture arrangement that helps with COPD and was shared between peers: “*This remedy relieves symptoms, and yet the doctor may not know of it*” (Pols 2012, p. 75). Across both conditions, participants indicated that without clinicians actually having their condition, they would not be able to fully comprehend what they were personally going through and what they were experiencing.

“I don’t think he gives the patient with diabetes the respect of which they deserve […], we’re living with it. It is very different from reading it from a book and saying ‘you should be doing this and you should be doing that.’ Practically, it doesn’t always work out.” - Diabetes Group Interview Participant

“I couldn’t handle the pain, so I took some of my old rescue (midrin) even though it hasn’t been that effective. I know I probably shouldn’t have without my doctors permission, but she isn’t the one that has to deal with the pain.” – Migraine Online Forum 1

The search for what others are experiencing happens in numerous and varied ways including interactions in person, such as attending support groups. However, these can be awkward to attend and infrequent, if they exist at all - diabetes has many more existing support groups compared to the migraine community. Support groups are a way for people to interact with their peers face-to-face and share common experiences in order to situate their illness experience and data. One diabetes group interview participant used the Internet to find out that having high blood sugar in the morning was not just something she experienced, but was common for others with diabetes and shared this with the group. Although a fellow participant who attends the same support group did not use the Internet to connect with peers, his attendance at these meetings meant that he was also able to compare this experience with that of other member’s to assess the normality of their common trigger.

Internet user: “I was panicking about that, and then I found lots of stuff on the internet about that so I felt…I felt it wasn’t just me!”

Fellow support group member: “Me too! In the morning!” – Diabetes Group Interview

Patients are increasingly going online for health information, and more frequently to connect with peers using Health 2.0 systems such as blogs and forums. Although this is not completely reliable medical information, participants suggested that their peers online provide information about real life experiences that can only be gained through actually living with the condition. The lack of reliability is not a deterrent for some participants as they see the value in reading about other people’s illness experiences to learn more about theirs.

“I think that yeah, because a lot of information is readily available on the net, and people are actually going out there and actually finding links to this through social networking and all the rest of it […]. And it definitely seems that people are in the know, they taught themselves. And they seem to know it very very well themselves.” – Diabetes Patient 2

People are supplementing clinical information by searching out others with similar symptoms, triggers, and treatments to better understand their condition and their experiences. They use this information to assess their self-care experiences, and some go even further by bringing the results of these searches back to their clinicians. Although an online forum member had access to general clinical information about migraines, they sought out more personal advice from peers in order to assess a personal symptom that a clinician could not give advice on, ultimately in order to try to gain a sense of what was normal for others with migraine.

“Has anyone ever experienced this? My question is am I alone in these long­lasting migraine/dizziness? And do a lot of people get severe dizziness with their migraines? […] Even my doctor seems at a loss. HELP.” – Migraine Online Forum 2

Of course this information seeking from a community of peers also occurs in the diabetes realm, but the nature and prevalence of the condition means that there are many more online sources, including the Diabetes Online Community (almost affectionately called the ‘D-Oh-C′ by members), a community network of health resources (Hilliard et al. 2015), including personal blogs that are well read and shared, according to the bloggers who were interviewed.

The need to situate their personal experiences within a frame of normality for their condition has led our participants to determined information seeking that can involve searching specifically for others with knowledge of their shared chronic condition and report similar health experiences. Despite issues with access, people are trying to compare their management practices with their illness peer group through patient generated information shared through various sources. This can happen in a number of ways such as through face-to-face interactions or over the Internet, but in most cases the information shared is not reliable medical information. This does not deter people from the practice as they are increasingly looking to the Internet and Health 2.0 tools (Kuehn 2013). Although people have information about themselves and their specific condition as well as medical information about the condition from their healthcare providers, our participants looked to varied and sometimes numerous peer-generated sources of information (e.g. support groups, forums, blogs, etc.) to validate their illness experience and assess their experience as ‘normal.’

## Discussion

Through the combined analysis of the data gathered about people’s information practices when managing two complex chronic conditions, we found similarities with regards to information seeking to help validate the experiences of living with the conditions. Participants look at their own personal health information, information about the condition in general, and what their peers have experienced. This information seeking contributes to a more grounded understanding of what is normal for them, what is normal for the condition, and what is normal for peers who are similar to them. The combination of these sources of information allows them to get closer to validating their illness experiences and answering the question ‘is my experience normal?’

In order for our participants to identify what was normal for them, they needed to know about their condition, where they fit in, and how others similar to them compared. For the chronic conditions of migraine and diabetes where there are many variables involved in symptoms, triggers, and treatments, our participants put substantial effort into understanding the condition in general, understanding the state of their own personal health, and understanding how this compares to their peers. Although there are commonalities in the amount of effort expended in this information seeking process, the participants used a variety of different means to collect this information, which they indicated can change over time. This process requires them to combine large, varied sets of information, both personal and generic, to understand normality within their condition and make sense of their personal illness experience compared to their peers.

Further to the complexity of combining the information, there are issues surrounding each of these information-seeking practices. We found that the personal nature and the number of variables that patients had to track could be overwhelming and lead to burn-out. Medical information was hard to gain from clinicians and hard to understand for laypeople. This led to patients seeking information from their peers in an inefficient way. Additionally, there were individual differences regarding preferences for information sources. The participants from both patient populations had their own preferences for tracking mediums (e.g. paper, computer spread sheets, smartphone apps, etc.), for seeking medical information (e.g. contact with clinicians, WebMD, etc.), and for getting peer support (e.g. support groups, forums, blogs, etc.). Our participants were primarily from urban and suburban areas in two countries, so we would expect that information seeking practices might vary even more significantly in different settings and in different countries.

Personal informatics to track personal information, medical information repositories to gain access to clinical information about a condition, and patient generated information to compare peer experiences all currently exist and are all useful in different ways to different people. The variety of apps available for aid the self-management of these conditions is evidence of this, and there are some tools such as Patientslikeme (Frost and Massagli 2008; Tempini 2015), that combine all three sources of information. However, most current tools concentrate on one or two of the necessary three ingredients for the assessment of what is normal that arose from our combined studies:

- knowledge of the self,
- knowledge of medical literature, and
- knowledge of others you can directly compare yourself to.

Additionally, many limit the person to using a strict template of what tracking mechanisms they can use, what medical resources they can access, and what peer support they allow. Each person has unique information needs and preferences, and existing tools do not support these differences. Like many other interactive technologies, they have limited flexibility in terms of what interactions they support. With these design decisions about the specific interactions that will be accommodated, we found they do not accommodate some of the more exploratory and serendipitous sources of information that participants had discovered, such as finding out somewhere on the internet that others experienced diabetes ‘morning highs’ and sharing this face-to-face with someone at a support group.

For diabetes and migraine where the conditions pervade everyday life and the onus of responsibility for condition management is on the patient, a ‘one size fits all’ technology may not be the answer. Storni identified the need for personalization of self-care technologies to get away from pre-defined technical solutions that do not encompass the complexity of self-care practices. He built an “open-ended editor that would allow bottom-up personalization of self-monitoring practices” (Storni 2011, p. 177) which, although quite flexible, still required the use of a mobile phone. Much of the work to develop self-care technologies presumes the use of a specific technology, increasingly mobile phones, as the best way to collect information about oneself, about medical information, or about what other people with the same condition are doing. However, our research shows that people’s preferred practices are far more idiosyncratic than what can be achieved by a single technology, such as an app. The current model of health informatics is top down and often jumps to the solution of apps for numerous and well-founded reasons, however this fails to accommodate the distinctive and personal preferences and needs uncovered by this research.

One possible approach to addressing these personal needs is to develop suites of tools that support different aspects and different variations of self-management information seeking, and that also work together seamlessly. This type of end user customisation could enable the individual to configure their own personal ‘ecology’ of tools that suits their needs at this time, recognizing that needs also evolve over time. For instance, migraine patients may be able to utilize a combination of mobile apps, online communities and other information resources including peers in social networks as a toolset for managing their conditions. However, due to the individual nature of each one’s own symptoms, triggers and unique situations, what constitutes this toolset requires one to recognize, discover, and reflect through their day-to-day disease management. This can be viewed as a Do-It-Yourself (DIY) health process since individuals have to identify their own set of systems, devices, and resources that work for themselves, and creating an ecology of tools that help them maintain a sense of normal. This DIY process relies on people living with health conditions, instead of designers or healthcare professionals. Although this will not suit every person self-managing a condition and engaging in exploratory search to validate illness experiences, tools such as Storni’s Tag-It-Yourself system (2011) might permit more flexibility in this endeavour without the responsibility or effort that comes with end-user customisation. Another possibility is that some people would be willing to take on even more responsibility for developing their own bespoke technologies and we can look towards the burgeoning open-source DIY communities to deliver novel bottom-up solutions.

There has been a recent push towards open source hardware in the medical domain (Schubert et al. 2014); within this community, people have started to discuss the development of bespoke medical devices to meet individual needs (Reynolds and Wyatt 2011; Niezen 2014; Vincent et al. 2015). In addition, there have been moves towards an open data approach to healthcare information, particularly in diabetes where information from medical devices is being transferred to the users through open software solutions^4^ and dedicated technologies.^5^ There is potential to build on these advances and tap into chronic condition communities to support bespoke hardware/software solutions that suit the individual in their information needs relating to self-management. This is already emerging in some places, such as the Google group MeDevice^6^ for people with diabetes who have an interest in hacking their devices or the #wearenotwaiting movement that helps people set up Nightscout, continuous glucose monitoring data on a variety of devices and smartwatches of their choice (Årsand et al. 2015). However, as this is an emerging trend, there are numerous concerns, issues and opportunities in DIY health and wellbeing that are only starting to be discussed by the CSCW and HCI research communities (O’Kane et al. 2016).

End user customisation and this burgeoning open-source trend could be leveraged for the information seeking needs of people who self-manage complex chronic conditions such as diabetes and migraine, to reduce the effort they have to put in to validating their illness experiences as ‘normal.’ With the variety of triggers, symptoms, and treatments they experience, it is not surprising that people with complex chronic conditions are seeking varied data sources to capture what they are personally going through, what the medical literature says, and what their peers are experiencing in order to understand what is normal around their illness experiences. Although there have been attempts to provide this variety of information, the solutions have been too narrow, with a restriction on the types of information sought or the sources of information that can be accessed.

Our studies show that for two communities of people with chronic illnesses that differ significantly in their manifestations and outcomes, there are striking similarities in people’s efforts to validate their experiences as ‘normal’. Future solutions need to provide this missing flexibility so that people can personalise their healthcare tools, choose their sources of information, and access their peers who have similar experiences.

## Conclusions

This paper presents findings on the information seeking needs and behaviours of people with complex chronic conditions trying to validate their illness experiences as ‘normal’. Although migraine and diabetes differ significantly from each other, they share important features in common; in particular, the idiosyncratic manifestation of these conditions makes it difficult for people to understand whether what they are experiencing is normal compared to their peers. The personal nature of managing health information, the effortful medical information seeking practices, and the move toward information seeking from peers highlight the need for combining current healthcare technologies that support each of these activities separately. We have highlighted the potential of personal health tools with the added functionality of flexible access to medical health repositories and peer generated repositories for alleviating some of the efforts that people with complex chronic conditions are currently putting into validating their experience. Future research could focus on allowing people to engage flexibly with health technology to help them understand their own condition, understand the condition in general, and understand what their peers are experiencing, giving them greater insight into what is ‘normal’ for them.

## Footnotes

1 “Diabetes is a chronic disease that occurs when the pancreas does not produce enough insulin, or when the body cannot effectively use the insulin it produces.” - http://www.who.int/diabetes/en/

2 “Migraine is a condition of recurring headaches that may be linked with other symptoms, such as sensitivity to light and noise, nausea, vomiting, dizziness and eyesight changes.” - http://www.migrainetrust.org/key-statistics

3
http://healthvault.com

4
http://tidepool.org/

5
http://diabe.to/

6
https://groups.google.com/forum/#!forum/medevice
